# Zulfiqar Frailty Scale: Overview, Stakes, and Possibilities

**DOI:** 10.3390/medicines8120073

**Published:** 2021-11-23

**Authors:** Abrar-Ahmad Zulfiqar, Ibrahima Dembélé

**Affiliations:** Service de Médecine Interne, Diabète et Maladies Métaboliques de la Clinique Médicale B, Hôpitaux Universitaires de Strasbourg et Equipe EA 3072 “Mitochondrie, Stress Oxydant et Protection Musculaire”, Faculté de Médecine-Université de Strasbourg, 67000 Strasbourg, France; ibrahimaadembele@gmail.com

**Keywords:** Zulfiqar Frailty Scale (ZFS), frailty syndrome, primary care

## Abstract

Very few frailty scales are used by general practitioners, as they are time consuming and cumbersome. We developed a frailty screening tool for use in primary care, referred to as the Zulfiqar Frailty Scale (ZFS). This scale was tested in multiple general practitioners’ offices in France, and these studies were published. In this paper, we offer a summary of these results.

## 1. Introduction and Development

According to the French National Institute of Statistics and Economic Studies (INSEE), 13.1 million people were aged 65 or older in France in 2018, or one in five inhabitants. By 2070, it is estimated that seniors will represent 29% of the French population. France’s elderly population has been growing steadily since 2011, or the year in which the first children of the baby boom generation turned 65. The year 2021 marks the end of the rise in 65–74 year-olds, as this is the year that all “baby boomers” will have reached the age of 65. The number of seniors aged 75–84 will increase from 2021 onwards, and from 2031 onwards for those aged 85 and over. Because of the increase in life expectancy, this aging of the population should continue until 2050 for 75–84 year-olds, and until 2060 for those aged 85 and over. After this time, the post-baby boom generations will contribute to a stabilization in the rise in the number elderly people in the population [1].

The stakes are high with regard to public health, as elderly people are at risk of chronic diseases, decompensation, a loss of autonomy, and (as a consequence) repeated recourse to care and hospitalization. As a society, we must therefore be proactive to prevent the healthcare system from becoming saturated and to improve the quality of life of patients. There are many different ideas as to what constitutes frailty in the elderly. Nevertheless, most literature reviews portray frailty as a dynamic and evolving concept that affects several aspects of daily life and may lead to a loss of autonomy [2]. Frailty cannot be reversed spontaneously. However, it can be reversed with targeted and proactive measures designed to bring about a reduction in morbidity and mortality [3].

General practitioners are often the first point of contact for care, and are therefore best suited to detect and implement the necessary actions to reverse frailty. There are many different scales for diagnosing frailty. However, these scales usually go beyond the scope of a general consultation. For example, the Fried Index (gold standard) requires the use of a dynamometer, a tool that is missing from most doctors’ offices. Moreover, sufficient space is required to allow patients to walk in a straight line (and thus measure their walking speed). As we can see from this example, certain scales are both technical and extremely time-consuming. Given the limited resources available to primary care providers, we decided to create a new frailty detection scale for use by general physicians. Unfortunately, these limited resources have become part of the undeniable and growing problem of frailty in the elderly, a major public health issue that is sure to remain problematic for many years to come. We developed a frailty screening tool that standardizes professional procedures and makes it possible for general practitioners to detect frailty in their elderly patients. This frailty scale (known as the “ZULFIQAR” scale) was created in an outpatient setting in conjunction with general physicians and tested for the first time in Plancoët in Brittany (Table 1). It includes six items which (as per the medical literature) are significantly and independently associated with a poor prognosis in terms of mortality and morbidity, and which can therefore be defined as frailty markers [4,5,6,7,8,9]. They were also chosen due to their simplicity and ability to be implemented quickly, since none of the items requires special equipment or prior training by the healthcare professional. The items were formulated as questions. A point is tallied for each positive response (“yes”). The patient is considered “non-frail” if their score is equal to 0, “not very frail” if their score is equal to 1 or 2, and “frail” if their score is greater than or equal to 3.

## 2. Main Results

Our frailty screening scale has been the subject of several published (or soon-to-be-published) studies since the original article was published in the journal MEDICINES MDPI. The results of the proof-of-concept study were very satisfactory and reproducible, and similar results have been found in subsequent trials. A simplified scale derived from the Zulfiqar Frailty Scale (sZFS) was created with five items (only one question regarding social interactions; the item “Does the patient benefit from home care?” was removed).

Table 2 below illustrates the main results of the various studies.

## 3. Discussion and Perspectives

In the various studies that were conducted, it took less than 2 min to complete the test. The scale is therefore ideal for an outpatient setting. Physicians are provided with all the necessary information on the patient’s treatments, social interactions, and nutrition. Each item in the questionnaire can potentially score one point, for a total of six points. The elderly person is considered “frail” by our ZFS scale if they score 3 or higher. For scores of 1 or 2, the patient is considered “pre-frail.” For a score of 0, the elderly person is considered “non-frail” or “robust.”

The results of the studies are very satisfactory: the correlations between the Zulfiqar Scale (and the simplified scale) and other frailty scales are very satisfactory.

In addition, the areas under the curve ranged from 0.80 to 0.94.

These results show that the ZFS and sZFS are outstanding tools for detecting frailty.

A future study will assess the ability of our ZFS frailty screening tool to predict the onset of morbidity and mortality within 6 months for a group of elderly subjects monitored by general practitioners, in particular with regard to falls, fractures, unscheduled hospitalizations (including emergency room visits), a loss of autonomy, institutionalization, and death. The study will begin in the Champagne-Ardenne and Normandy regions of France. 

Our scale could also be used in doctors’ offices and multidisciplinary clinics, where the detection of frailty can be accompanied by targeted measures thanks to the many healthcare professionals present. Considering the shortage of frailty clinics and geriatricians, this could prove extremely beneficial. Naturally, general physicians will be able to create personalized treatment plans and play a central role in the care of their patients. They will coordinate with paramedical and social professionals, reassess care options, and help manage family caregivers. It is estimated that 30% to 40% of elderly people living at home are frail. 

Given the scope of this syndrome and (in certain areas) the shortage of qualified physicians, local paramedical professionals (private nurses, physiotherapists, pharmacists, etc.) must also be able to screen for frailty. We have therefore set up a website that can be accessed by all medical professionals (as well as paramedical professionals such as home care nurses, occupational therapists, and physical therapists) from anywhere. This website is available at the following link: http://zulfiqarfrailtyscale.com/ (accessed on 1 June 2021). A mobile app will also be released soon.

## Figures and Tables

**Table 1:** Zulfiqar Frailty Scale: ZFS.

Cotation	1	0
Is there a weight loss greater than or equal to 5% in 6 months?	Yes	No
Monopod support test < 5 s?	Yes	No
Does the person live alone?	Yes	No
Does the patient receive home care?	Yes	No
Does the person complain of memory loss?	Yes	No
Does the person have prescriptions for more than 5 therapeutic classes on his/her prescription history for at least 6 months?	Yes	No
**Total/6**	**=0: none** **=1–2: pre frail** **≥3: frail**	

**Table 2:** Summary of the characteristics of the different studies and populations.

	Creation of a New Frailty Scale in Primary Care: ZFS [5]	Frailty in Primary Care Validation of the sZFS * [10]	Validation of the Zulfiqar Frailty Scale (ZFS): A New Tool for General Practitioners [11]	Validation of a New Frailty Scale in Primary Care: The sZFS * [12]	Creation of a New Frailty Scale in Primary Care: The ZFS * [13]
Populati on	≥75 y, ADL * ≥ 4	≥65 y, ADL * ≥ 4	≥65 y, ADL * ≥ 4	≥65 y, ADL * ≥ 4	≥75 y, ADL * ≥ 4
Localization	Brittany	Normandy	Alsace	Champagne-Ardennes	Poitou-Charente
Inclusion period	1 November 2018– 30 April 2019	1 November 2017– 1 April 2018	1 November 2020– 30 April 2021	2 May 2019– 30 April 2020	1 May 2019– 30 May 2020
Number of elderly patients included	102 patients	107 patients	102 patients	268 patients	200 patients
Male	47	43	47	143	97
**Female**	**55**	**64**	**55**	**125**	**103**
Age	82.65 (4.79)	74 (7)	75.9 (8)	77.5 (7.8)	81.4 (4.82)
ADL */6	5.39 (0.73)	5.87 (0.34)	5.83 (0.35)	5.60 (0.90)	5.81 (0.53)
IADL */8	5.52 (2.57)	7.65 (0.85)	7.04 (1.68)	0.72 (1.08) (IADL/4)	6.19 (1.58)
Charlson comorbidities index	3.08 (1.87)	4.38 (1.99)	4.11 (1.81)	2.43 (1.92)	4.63 (1.01)
Drugs number	6.37 (2.78)	5.1 (2.9)	4.3 (3.0) (Therapeutic class)	7.59 (3.84)	6.32 (3.6) (Therapeutic class)
Compared with	Fried Modified SEGA * scale GFST * scale CGA *	GFST * scale	Modified SEGA scale	Fried	Fried
Duration time ZFS */sZFS * (seconds)	109.62	77	92.75	110	71.7

* ADL: activity of daily living; y: years; sZFS: simplified Zulfiqar Frailty Scale; ZFS: Zulfiqar Frailty Scale; GFST: Gérontopôle Frailty Screening Tool; SEGA: Short Emergency Geriatric Assessment; IADL: Instrumental Activity of Daily Living; CGA: Comprehensive Geriatric Assessment. Data expressed as: mean (standard deviations).

## Data Availability

Not applicable.
